# A90 IMPLEMENTATION OF NATIONAL GUIDELINES ON THE MANAGEMENT OF VACCINE PREVENTABLE ILLNESS IN PATIENTS WITH INFLAMMATORY BOWEL DISEASE: PERCEIVED BARRIERS AND INTERVENTION FUNCTIONS AMONGST GASTROENTEROLOGISTS

**DOI:** 10.1093/jcag/gwac036.090

**Published:** 2023-03-07

**Authors:** F Zhou, J Robar, M Stewart, J Jones

**Affiliations:** 1 Dalhousie University; 2 Nova Scotia Health Authority, Halifax, Canada

## Abstract

**Background:**

Vaccination uptake amongst patients with IBD remains suboptimal. Studies evaluating effectiveness of interventions designed to improve vaccine uptake have not assessed perceived barriers and solutions related to implementation of evidence-based guidelines for vaccine preventable illness (VPI).

**Purpose:**

The aim of this study was to identify barriers and facilitators for evidence-based management of VPI in IBD.

**Method:**

A semi-structured interview was conducted with gastroenterologists. Interview questions were developed and guided by the COM-B and TDF evidence-based implementation science frameworks. A brief intake questionnaire was administered to collect participant demographic and clinical practice information. Gastroenterologists were recruited through direct local contact via email by the investigators. Sixty minute interviews were recorded and transcribed for data analysis. Using thematic analysis, codes from the study data will be generated to identify themes. The data will be categorized into the coding scheme and themes created using an inductive coding approach.

**Result(s):**

As of October 2022, 5 interviews were conducted. Mean participant age was 47.8 years, with 60% identifying practice in an urban/academic setting compared to a rural/community setting (20%). Preliminary major themes included 1) assessing vaccination status and recommending appropriate vaccines are the responsibility of the gastroenterologist 2) gastroenterologists need more support to administer vaccines in clinical practice 3) barriers to implementation of VPI guidelines include lack of access to a family physician, limited time, vaccine hesitancy, and incomplete understanding of coverage/access to vaccines and 4) intervention themes include use of clinical decision support tools embedded into the workflow of healthcare providers, need for support from allied healthcare providers, increased need for third party support, and more education/CME relating to management of VPI in clinical practice. Specific knowledge gaps include 1) uncertainty relating to what vaccines are covered financially 2) lack of knowledge of risk factors for specific VPI such as pneumococcus and meningococcus and 3) how to administer live vaccines in patients already on immunosuppressants.

**Conclusion(s):**

Preliminary qualitative themes suggest that although gastroenterologists acknowledge the importance of managing VPI in patients with IBD, perceived resource, policy, and educational barriers exist. The qualitative data from this study will be used to design and implement customized, evidence-based implementation strategies for managing VPI that are sensitive to the local environment.

**Please acknowledge all funding agencies by checking the applicable boxes below:**

None

**Disclosure of Interest:**

None Declared

